# How do breastfeeding workplace interventions work?: a realist review

**DOI:** 10.1186/s12939-021-01490-7

**Published:** 2021-06-25

**Authors:** Kathrin Litwan, Victoria Tran, Kate Nyhan, Rafael Pérez-Escamilla

**Affiliations:** 1 Department of Social and Behavioral Sciences, Yale School of Public Health, Yale University, 47 College Street, New Haven, CT 06510 USA; 2 Harvey Cushing / John Hay Whitney Medical Library, Yale University, New Haven, CT USA; 3 Department of Environmental Health Sciences, Yale School of Public Health, Yale University, New Haven, CT USA

**Keywords:** Breastfeeding, Breastfeeding support, Lactation support, Work, Worksite, Workplace, Lactation program, Policy

## Abstract

**Background:**

Women are representing an increasing share of the labor force, thus, raising the need to accommodate breastfeeding working mothers at the workplace. While there is an emerging body of evidence supporting the positive influence of workplace lactation programs on breastfeeding outcomes, there is a lack of literature on the mechanisms underlying those interventions. Aims of this realist review were three-fold: to uncover underlying mechanisms, determine who benefits the most from such interventions and important contextual factors influencing uptake.

**Methods:**

Purposive bibliographic searches on Medline, Web of Science Core Collection, CINAHL, Global Health, LILACS, Global Index Medicus, Business Source Complete, Proquest Dissertations and Theses and Open Access Theses and Dissertations were conducted to identify relevant publications. Included publications (qualitative and quantitative) described interventions aiming to improve the breastfeeding behavior of working mothers, that were initiated by the employer, reported on breastfeeding outcomes and had a clearly defined workplace. Publications only focusing on maternity leave or that were not published in English, Spanish, Portuguese or German were excluded. A realist approach was followed to identify how workplace interventions work, who benefits the most and the important contextual factors.

**Results:**

The bibliographic search yielded a total of 4985 possible publications of which 37 publications were included in the realist analysis. Effective workplace breastfeeding interventions activate three mechanisms: 1) awareness of the intervention, 2) changes in workplace culture, manager/supervisor support, co-worker support and physical environments, and 3) provision of time. Contextual factors such as the distance between the workplace and the infant and the type of workplace may influence the degree of activation of the underlying mechanisms for programs to positively impact breastfeeding outcomes.

**Conclusions:**

In order to be effective, workplace breastfeeding interventions need to: raise awareness of the intervention(s) available among working mothers as well as their work environment, change the workplace culture, foster manager/supervisor support and co-workers support, provide enough time and adequate space and facilities for women to breastfeed or express breastmilk during the workday.

**Supplementary Information:**

The online version contains supplementary material available at 10.1186/s12939-021-01490-7.

**Table:** 

This article is a part of the Interventions and policy approaches to promote equity in breastfeeding collection, guest-edited by Rafael Pérez-Escamilla, PhD and Mireya Vilar-Compte, PhD	

## Background

In the last decades, the number of women entering the labor force has steadily increased and women are representing a larger share of the labor market than ever before [1]. Globally, women’s labor force participation rate is 48.5%, which demonstrates their growing and significant contribution to their various national economies [2]. Because of women’s growing presence in the labor market, there is an increasing number of employers seeking to accommodate the needs of working women who choose to have children and want to breastfeed. Indeed, employers already recognizing the importance of breastfeeding, have offered various levels of lactation support especially for women working in the formal but not the informal sector [3, 4].

It is well established that breastfeeding is associated with numerous short- and long-term health benefits for the breastfeeding mother and the breastfed child. As such, breastfed children have lower risk for morbidity and mortality from infectious diseases, increased intelligence scores, and a reduction in risk for overweight and perhaps diabetes in later life [5–7]. For mothers, breastfeeding is associated with lower risk for breast cancer, ovarian cancer and type 2 diabetes [8].

Despite the various benefits of breastfeeding and the WHO/UNICEF recommendation for exclusive breastfeeding for 6 months, globally only about 44% of infants < 6 months of age were exclusively breastfed in 2019 [9]. This is still below the goal of at least 50% by 2025 defined in the Global Nutrition Targets 2025 by UNICEF and WHO [10]. Maternal employment is often cited as a major barrier to breastfeeding [11] and returning to work is associated with early cessation of breastfeeding [12, 13].

Previous research has shown a positive association between workplace lactation support and interventions with higher breastfeeding rates and duration of breastfeeding [14]. Relatively low-cost interventions such as lactation rooms and nursing breaks may reduce absenteeism and improve workplace performance, commitment and retention, while also improving breastfeeding outcomes [11, 15]. Also, mothers receiving other types of workplace support such as provision of electric breast pumps, access to lactation professionals, and refrigerators for storing their breastmilk in the workplace were more likely to initiate and continue breastfeeding after returning to work [14, 16]. Availability of employer-sponsored childcare and flexible schedules can also increase an employee’s likelihood of success with breastfeeding [17]. Therefore, more and more countries and organizations are introducing measures to support working mothers in reaching their breastfeeding goals [18], thus, enabling mothers to better combine their work requirements and their infant feeding goals.

While there is an emerging body of evidence supporting the positive influence of workplace lactation programs on breastfeeding outcomes [14–16, 19], there is a lack of literature on the mechanisms underlying those interventions. An understanding of these mechanisms is crucial in learning how to operationalize, implement and disseminate robust and effective lactation programs. Systematic reviews focus on the outcome and the level of outcome, thus on statistical inferences, but do not investigate the underlying mechanisms or context of the intervention leading to those outcomes. However, in order to find how interventions work in different contexts, statistical inferences are insufficient. Disentangling the underlying mechanisms linking intervention, context and outcome are needed to support the policymaking and implementation processes under ‘real world’ conditions. Thus, in addition to probability considerations, plausibility and adequacy considerations [20] need to be taken into account in order to understand how workplace-based breastfeeding interventions actually work.

Given the need for an approach that accounts for context, the objective of this study was to follow a realist approach to better understand how worksite related breastfeeding programs work across different contexts. We followed the realist review approach as it does not focus on making statements about the strength of quantitative vs. qualitative study designs but rather it integrates and values the different perspectives offered by them and enable the researcher to unravel and understand underlying pathways. Furthermore, workplace breastfeeding interventions meet the seven criteria of complex service interventions that can best be examined through the lens of a realist review [21] (Table 1).

**Table 1:** The 7 Criteria of Complex Service Interventions and Their Evaluation Through Realist Reviews (after [21])

Criteria of complex service intervention and their evaluation through realist reviews	Application to workplace breastfeeding programs
1) Public health interventions hypothesize that after implementing an intervention, the condition will be improved. The review needs to closely follow those underlying theories.	Workplace breastfeeding programs are expected to improve breastfeeding rates among participating working mothers.
2) Public health interventions only accomplish their goals by active input from individuals. It is therefore essential to not only rely on controlled study designs but to also review the actions of involved stakeholders to be able to explain the success or failure of the intervention.	In order to have a successful workplace breastfeeding intervention, active input from several individuals is needed e.g. communicate the availability of a breastfeeding/lactation room, active support from co-worker to take over responsibility while the breastfeeding women is on her pumping break, etc.
3) Public health interventions involve a long and complex pathway which includes the operational changes needed to implement the intervention and the uptake and adherence to the program by the participants. Thus, the review needs to consider the entire implementation chain, determine the needed intermediate outputs for a successful final outcome and define processes and blockage points by including a variety of publication types.	In order to understand workplace breastfeeding interventions, the review needs to analyze who initiated the program, how it was developed, how it was implemented and who actively participated in the implementation and how as well as who adheres to the offered breastfeeding intervention.
4) Public health interventions are non-linear. The review therefore needs to determine and account for the different influences of the different parties by not only including controlled trials.	Within the implementation chain of a workplace lactation program, different stakeholders influence each other. Depending on their power, the workplace breastfeeding program differs e.g. workplace lactation programs might be more extensive if employee have strong representatives compared to situations in which the employers strictly follow governmental regulations.
5) Public health interventions are embedded in multiple social systems. Such differences are best uncovered by considering finding from qualitative study designs.	The wish for privacy in a lactation room in a woman dominated environment may be different then in a male dominated work environment.
6) Public health interventions differ depending on the context and the understanding of the implementing stakeholder. The review needs to help to understand what similar terms mean to different stakeholders by disentangling underlying implementation mechanisms and contexts behind interventions labelled the same way but in reality being different from each other.	A workplace lactation room can range from simply referring to an empty room vs. a fully equipped lactation room with an accompanying policy regulating breaktime and flexible working hours.
7) Public health interventions are open systems with feedback loops. Hence changes in overall environment as a result of interventions need to be considered across different contexts.	By successfully implementing a workplace lactation policy, the overall work-based environment may change and as a consequence, the policy itself strengthens.

In order to inform policy makers and employers about workplace breastfeeding interventions, the specific questions we aimed to answer though this realist review were: 1) *How* do breastfeeding interventions at the workplace work?, 2) *Who benefits* the most from such interventions across different contexts?, and 3) *What are important contextual factors* which determine whether different mechanisms produce their intended breastfeeding outcomes?

## Methods

A protocol was written and made publicly available a priori at https://osf.io/phndm/. Due to time limitations, grey literature searches as well as citation chaining as described in the protocol were not conducted.

### Search methods and criteria for identification of studies

The search of the bibliographic databases was conducted by a medical research librarian. Controlled vocabulary and keywords for the two concepts “breastfeeding” and “workplace” were used for the search of the bibliographic databases in order to achieve high specificity (Table 2). In databases without subject indexing, we achieved high specificity by searching for the concepts of breastfeeding and workplace in titles and author-provided keywords. The Medline search is provided in Table 3; the remaining searches are provided in Additional file 1.

**Table 2:** Overview of Bibliographic Databases and Platforms Used in the Search Process. Realist Review on “How Do Breastfeeding Workplace Interventions Work?”

• Medline (Ovid) • Web of Science Core Collection, as licensed at Yale University: • Science Citation Index Expanded (SCI-EXPANDED) --1900-present • Social Sciences Citation Index (SSCI) --1900-present • Arts & Humanities Citation Index (A&HCI) --1975-present • Conference Proceedings Citation Index- Science (CPCI-S) --1991-present • Conference Proceedings Citation Index- Social Science & Humanities (CPCI-SSH) --1991-present • Book Citation Index– Science (BKCI-S) --2005-present • Book Citation Index– Social Sciences & Humanities (BKCI-SSH) --2005-present • Emerging Sources Citation Index (ESCI) --2015-present • CINAHL (Ebsco) • Global Health (Ovid) • LILACS • Global Index Medicus • Business Source Complete (Ebsco) • Proquest Dissertations and Theses • Open Access Theses and Dissertations

**Table 3:** Medline Search Strategy. Realist Review on “How Do Breastfeeding Workplace Interventions Work?”

Ovid MEDLINE(R) ALL < 1946 to October 01, 2020>		
Line	Query [comments in square brackets]	Results
1	[Kathrin Litwan project]	0
2	[medline]	0
3	[breastfeeding concept]	0
4	exp breast feeding/	38,035
5	(breastfe* or breast-fe*).mp.	58,767
6	lactation.mp.	60,214
7	breast pump*.mp.	389
8	(express* adj2 milk).mp.	1604
9	[workplace context concept]	0
10	workplace/	23,180
11	Employment/ or work/ or “personnel staffing and scheduling”/	81,730
12	women, working/	5375
13	return to work/	2629
14	job satisfaction/	25,055
15	work schedule tolerance/	6918
16	[tight focus on breastfeeding]	0
17	3 or 4 or 5 or 6 or 7 or 8	110,302
18	[for Covidence upload and screening, this is a set of papers with an explicit breastfeeding reference and EITHER workplace indexing OR *if* they haven’t yet been indexed, some form of the word “work” in the title or author keywords]	0
19	10 or 11 or 12 or 13 or 14 or 15	132,080
20	work*.ti,kf. not medline.st.	38,425
21	17 and (19 or 20)	1007

The results from the search of all bibliographic databases were deduplicated in EndNote X9 and imported to Covidence systematic review software (Veritas Health Innovation, Melbourne, Australia. Available at www.covidence.org). Both software packages were used in the versions licensed at Yale University.

In order to be included, the publications must have described interventions that aimed to or could be expected to improve the breastfeeding behavior of working mothers and that were initiated by the employer, its representative, an employer-like persona or the work supervisor of the parent(s). Furthermore, the study must have reported on breastfeeding outcomes (quantitative or qualitative) and the workplace must have been clearly defined (either real or virtual space). Any data that described breastfeeding behavior were considered as breastfeeding outcomes. Therefore, studies reporting on quantitative breastfeeding outcomes (e.g. exclusive breastfeeding rate, exclusive breastfeeding duration, breastfeeding cessation, duration of any breastfeeding) as well as on qualitative breastfeeding outcomes were included. No date limit for publications nor limitation on types of data collected in the study (qualitative vs. quantitative data) were applied as inclusion or exclusion criteria.

Publications that only focused on maternity leave and that were not published in English, Spanish, Portuguese or German were excluded. Publications only focusing on maternity leave interventions were excluded because the review’s focus is workplace breastfeeding interventions that can be fully influenced by the employer. Maternity leave interventions are often regulated, at least to some extend by governments, and are thus, not under the sole control of the employer. Publications that were published in languages other than English, Spanish, Portuguese or German are listed in a separate supplementary table (Additional file 2). Literature reviews were excluded from the analysis.

The inclusion criteria as well as the maternity leave exclusion criteria were applied to the title-abstract screening stage. Articles initially selected for full-text screening were screened in detail for all inclusion and exclusion criteria. Exclusions at the full-text screening stage were grouped into following reasons: 1) intervention was not initiated by the employer, 2) breastfeeding outcomes not reported, 3) article focused on maternity leave, 4) article not published in English, Spanish, Portuguese or German, and 5) other reasons. All screening rounds were conducted by two reviewers following a consensus approach (KL and VT). Discrepancies were resolved through discussion and consultation with a third reviewer (RPE).

Non-peer-reviewed documents (e.g., conference papers, press releases, etc.) were not included. When such documents were retrieved in the database searches, the document’s authors were contacted for more information when deemed necessary. Dissertations were handled as peer-reviewed articles and authors were not contacted.

Goal of the data extraction was to find context-mechanism-outcome (CMO) patterns that can potentially explain the relationship between the intervention and the outcome by understanding the underlying mechanism in the context in which the intervention takes place. Focusing the data extraction on contexts, mechanisms and outcomes is important to identify reasons behind program successes or failures instead of just identifying successful and unsuccessful interventions [21]. Data extraction was conducted by the two reviewers. The data points extracted from peer-reviewed articles were: year of publication, type of study/publication, country, implementation (type, components/activities and approach), context (full-/part-time employment, type of work position, length of maternity leave, etc.), sample size, implementation period, intervention population and implementation outcomes (acceptability, adaptation, appropriateness, cost, feasibility, fidelity, penetration and sustainability). Additionally, breastfeeding outcomes were extracted to better understand the influence of the intervention on breastfeeding outcomes.

For the development of a CMO framework, we defined the intervention types, intervention category, context categories as well as the breastfeeding outcome for each included study. The CMO framework and findings were summarized and discussed in a narrative synthesis.

## Results

### Description of articles found through bibliographic search

**Fig. 1:**
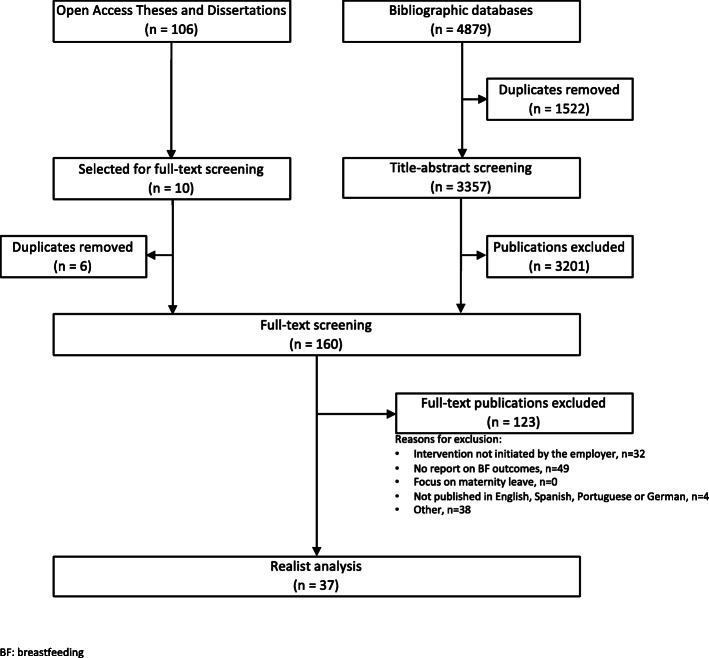
Overview of Search and Screening Process. Realist Review on “How Do Breastfeeding Workplace Interventions Work?”

The bibliographic database search yielded a total of 4879 possible documents. After removing a total of 1522 duplicates, 3357 citations were screened at the title-abstract phase in Covidence. From these 3357 citations, 156 were eligible for full-text screening. Separately, the search on Open Access Theses and Dissertations (OATD) resulted in a total of 106 possible documents. Because of technical issues with the exportation of search results, the 106 documents from OATD were handled outside of Covidence during the title-abstract screening, thus, only one reviewer screened the OATD search results. A total of 10 OATD documents were found to be eligible for the full text screening. After removing 6 documents that had already been screened in Covidence as a result of the other searches, 4 OATD documents were imported into Covidence for the full-text screening. This led to a total of 160 articles that were eligible for the full-text screening which was conducted by two reviewers. At the full-text screening phase a total of 123 articles were excluded, leaving 37 articles for analysis. Articles were excluded for the following reasons: intervention was not initiated by the employer (32 articles), no report of breastfeeding outcomes (49 articles), not published in English, Spanish, Portuguese or German (4 articles) or for other reasons (38 articles). Subcategories under “other reasons” included articles which could not be delivered via interlibrary loan (10 articles), literature reviews (7 articles) and duplications discovered during full text screening (2 articles). Only one reason for exclusion could be recorded in Covidence. An overview of the screening process is depicted in Fig. 1.

The 37 articles that were included in the realist analysis came from 11 countries. The majority of publications were from studies conducted in the United States of America (19 articles [22–40];). The other publications came from studies in Taiwan (5 articles [41–45];), Brazil (4 articles [46–49];), Thailand (2 articles [50, 51];), Australia [52], China [53], Ethiopia [54], Indonesia [55], Mexico [56], Spain [57] and Turkey [58] (each 1 article). The 37 articles resulted from 33 single studies published over a period of 35 years; from 1985 to 2020. An overview of the included studies is given in Tables 4, 5, 6, 7, 8 and 9.

**Table 4:** Summary of Publications Included in the Realist Analysis Following a Case-Control Design. Realist Review on “How Do Breastfeeding Workplace Interventions Work?”

CASE-CONTROL DESIGN	
Lead Author; Publication Year	Sahip et al. [58]; 2007
Study Population	Expectant fathers of companies with full-time workplace physician Cases (*N* = 80): men’s characteristics: 32.5% < 30 years of age, 67.5% university education, 81.3% first child; wife’s characteristics: 60.0% < 30 years of age, 60.0% university education, 53.8% working outside the home Controls (*N* = 80): men’s characteristics: 31.3% < 30 years of age, 67.5% university education, 70.0% first child; wife’s characteristics: 60.0% < 30 years of age, 45.0% university education, 48.8% working outside the home NS differences between cases and controls
Country	Turkey
Intervention	6 education sessions each 3–4 h for expectant fathers: health during pregnancy, pregnancy nutrition + birth, communication techniques, infant health care and feeding, fatherhood, family health after birth
Outcomes	BF initiation within 1 h after birth: OR = 2.38, 95% CI: 1.24–4.61, *p* < 0.01 EBF at 3 months: OR = 3.44, 95% CI: 1.74–6.82, *p* < 0.01 BF at 9 months: OR = 2.64, 95% CI: 1.36–5.09, *p* < 0.01 Supplementary feeding before 6 months: OR = 0.19, 95% CI: 0.09–0.37, *p* < 0.01
Lead Author; Publication Year	Waite et al. [22]; 2015
Study Population	Women with children and employed with same employer after return to work; Case company (*N* = 131): Large corporation in the US Southeast with lactation program; mean age 33.2 ± 3.8 years; 81.8% White, 8.1% Asian, 2.5% Black or African American, 7.6% other; 96.3% married or living with partner; 15.9% less than college graduate, 50.4% 4-year college, 33.7% advanced degree; Control company (*N* = 420): Seattle Children’s Hospital; mean age 34.5 ± 2.9 years; 81.3% White, 8.6% Asian, 4.7% Black or African American, 5.4% other; 96.9% married or living with partner; 3.9% less than college graduate, 53.5% 4-year college, 42.6% advanced degree
Country	USA
Intervention	N/A
Outcomes	BF initiation: 100% (case) vs. 98% (control) Mean BF duration: 38.8 ± 34.1 weeks (case) vs. 41.8 ± 24.0 weeks (control) BF at 6 months: 60% (case) vs. 79% (control) Mean total support score: 137.7 ± 15.1 (case) vs. 124.5 ± 14.9 (control) NS association between support scores and breastfeeding durations
Lead Author; Publication Year	Lin et al. [41]; 2020
Study Population	Case companies (*N* = 1089): Companies accredited as healthy workplaces under Tobacco Control and/or Occupational health Promotion legislation from 2007 to 2008; Control companies (*N* = 526): Companies without accreditation, randomly selected from the National Business Directory; For breastfeeding outcome: women who returned to work; *N* = 477
Country	Taiwan
Intervention	Lactation break times Availability of lactation policy or documentation BF promotion Provision of lactation rooms Provision of refrigerator to store expressed BM Provision of child-care facility
Outcomes	Continuing BF after 1 year: • AOR = 3.32, 95% CI:1.90–5.77 if break times are provided • AOR = 2.50, 95% CI:1.59–3.92 if BF policy or documentation is available • AOR = 2.25, 95% CI: 1.45–3.48 if BF is promoted • AOR = 3.00, 95% CI: 1.89–4.76 if lactation rooms are provided • AOR = 2.35, 95% CI: 1.23–4.45 if refrigerators are provided • AOR = 2.58, 95% CI: 1.40–4.75 if child-care facility is available
Lead Author; Publication Year	Hilliard et al. [32]; 2020
Study Population	Working women in North Dakota who attempted to continue BF after returning to work outside the home following a birth of a child between 2014 and 2016; Mean maternal age: 30.8 ± 4.1 years; Maternal race: 97.0% White, 0.6% Black, 0.2% Asian/Pacific Islander, 1% Native American/Alaskan Native, 0.6% mixed race, 0.6% declined; Marital status: 94.0% married, 4.3% cohabitating, 1.0% single, 0.7% other; Income: 0.3% < USD 15,000, 1.1% USD 15,000-24,999, 3.1% USD 25,000-34,999, 6.8% USD 35,000-49,999, 18.0% USD 50,000-74,999, 29.9% USD 75,000-99,999, 40.8% ≥ USD 100,000; Maternal education level: 1.6% high school, 9.1% some college, 12.2% associate degree, 42.0% Bachelor’s degree, 6.1% some graduate, 29.0% graduate degree; *N* = 392
Country	USA
Intervention	Infant-friendly business designation incl.: • Workplace lactation policy • Allowance of flexible break times • Provision of private space (other than bathroom) with a source of clean water to wash hands Provision of refrigerator for BM storage
Outcomes	BF duration according to designation status: • Total mean duration: 8.62 ± 4.89 months • Designation in 2011 or 2012 and recent recertification: 7.97 ± 5.60 months • Designation later than 2012: 7.69 ± 4.98 months • Designation in 2011 or 2012 but no recertification: 6.07 ± 4.32 months • No designation: 9.00 ± 4.68 months *p* = 0.30
Lead Author; Publication Year	Cervera-Gasch et al. [57]; 2020
Study Population	Female teachers/researchers or administration/service staff at either Universitat Jaume (UJI) or Universidad de Sevilla (US) who gave birth in the past 10 years and were employed at either UJI or US at the time of delivery and/or BF; Universitat Jaume (case): *N* = 103 Maternal education level: 1% secondary education, 99% university studies Universidad de Sevilla (control): *N* = 198 Maternal education level: 1% primary education, 11.1% secondary education, 87.9% university studies
Country	Spain
Intervention	Universitat Jaume (UJI) • 4 designated BF areas • BF education Universidad de Sevilla (US) • No lactation rooms • No lactation support program
Outcomes	Intention to BF: 93.2% (UJI) vs. 88.4% (US), *p* = 0.051 Intention to continue BF after RTW: 77.7% (UJI) vs. 66.7% (US), *p* = 0.580 Continued BF after RTW: 71.8% (UJI) vs. 50.5% (US), *p* = 0.001 BF duration • UJI: 15.5% < 6 months, 13.6% 6–12 months, 28.2% 1–2 years, 32.0% > 2 years • US: 39.9% < 6 months, 18.2% 6–12 months, 16.7% 1–2 years, 10.6% > 2 years • *p* < 0.001

*95% CI* 95% confidence interval, *AOR* addjusted odds ratio, *BF* breastfeeding, *BM* breastmilk, *EBF* exclusive breastfeeding, *N/A* not applicable, *NS* not significant, *OR* odds ratio, *RTW* return to work

**Table 5:** Summary of Publications Included in the Realist Analysis Following a Cohort Design. Realist Review on “How Do Breastfeeding Workplace Interventions Work?”

COHORT DESIGN	
Lead Author; Publication Year	Brasileiro et al. [46]; 2012
Study Population	Formally working mothers who RTW before the child had reached six months of life; Maternal age: 52.0% ≤28 years; Paternal age: 53.0% ≤30 years; Marital status: 94.5% with partner; Socioeconomic level: 14% high, 38.0, 12.4% low; *N* = 200
Country	Brazil
Intervention	Daycare center Lactation facilities
Outcomes	Discontinue BF before 4 months: • Not having a 30-min break every work shift: OR = 4.10, 95% CI: 1.81–9.26, *p* < 0.0001 • NS difference for presence of daycare center at the workplace • NS for number of working hours per day • NS for presence of lactation facilities

*95% CI* 95% confidence interval, *BF* breastfeeding, *NS* not significant, *OR* odds ratio, *RTW* return to work

**Table 6:** Summary of Publications Included in the Realist Analysis Following a Cross-Sectional Design. Realist Review on “How Do Breastfeeding Workplace Interventions Work?”

CROSS-SECTIONAL DESIGN	
Lead Author; Publication Year	Jacknowitz [40]; Chapter 4; 2004
Study Population	National Longitudinal Survey of Youth 1979, analysis is limited to birth between 1989 and 1999; Birth mother must have worked ≥20 h per week during six months prior to birth; Maternal age: 31.4 ± 3.34 years; Maternal race: 56.7% Non-Hispanic White, 23.8% Non-Hispanic Black, 19.5% Hispanic; Marital status: 82.9% husband/partner present; BF initiation: 59.6%; Any BF rate at 6 months: 19.5%; *N* = 893
Country	USA
Intervention	Employer-sponsored childcare Flexible work schedule Hours worked at home
Outcomes	Employer-sponsored childcare • BF initiation: NS • BF at 6 months: 11.4% higher probability of BF at 6 months with employer-sponsored childcare than without, *p* < 0.01 Flexible schedule • BF initiation: NS • BF at 6 months: NS Hours worked at home • BF initiation: every 8 h worked at home per week is associated with 0.7% higher probability of BF initiation, *p* < 0.05 • BF at 6 months: every 8 h worked at home per week is associated with 0.5% higher probability of BF at 6 months, *p* < 0.01 Shift work • BF initiation: NS • BF at 6 months: NS
Lead Author; Publication Year	Ortiz et al. [39]; 2004
Study Population	Participants of corporate lactation programs of five companies who gave birth between April 19, 1993 and December 31, 1997 and full-time employed before taking maternity leave; Maternal race (only 4 of the 5 companies provided demographic data): 0.6% American Indian, 14.1% Asian/Pacific Islander, 6.2% African American, 26.8% Hispanic, 52.3% White; *N* = 462
Country	USA
Intervention	Corporate management policies guaranteeing that BF employees will be supported Private, locked rooms for pumping Provision of electric breast pump Breast pump instructions BF class BF education and lactation consultations BF telephone consultations 24/7
Outcomes	BF initiation: 97.5% Any BF rate at 6 months: 57.8% Pumped at work: 152 of 194 (salaried) vs. 104 of 157 (hourly), *p* ≤ 0.01 Mean age of infant when pumping was discontinued: 9.1 ± 4.11 months • 9.0 ± 4.26 months (full-time) vs. 8.6 ± 2.95 (part-time), *p* = 0.72 • 8.7 ± 3.92 months (salaried) vs. 9.3 ± 4.51 (hourly), *p* = 0.31
Lead Author; Publication Year	Chen et al. [44], 2006
Study Population	Female employees of Company T who have taken maternity leave between January 1999 and April 2003; Maternal age: 6.6% 20–24 years, 42.0% 25–29 years, 40.7% 30–34 years, 10.7% ≥35 years; Mean maternal age: 29.8 ± 3.7 years; Maternal education level: 51.6% ≤ high school, 48.2% ≥ college; Worksite: 82.1% clean room; Shift work: 77.2% yes; Flextime: 29.4% yes; *N* = 998
Country	Taiwan
Intervention	Lactation rooms Lactation break times
Outcomes	OR for ever breastfed: • Awareness of lactation room (yes vs. no): 1.60, 95% CI: 1.14–2.24, *p* = 0.006 • Awareness of lactation breaks (yes vs. no): 0.87, 95% CI: 0.59–1.28, *p* = 0.474 • Awareness of lactation room among clean room workers (yes vs. no): 7.88, 95% CI: 2.36–26.32, *p* = 0.001 • Awareness of lactation breaks among clean room workers (yes vs. no): 0.84, 95% CI: 0.56–1.26, *p* = 0.399 • Awareness of lactation room among office workers (yes vs. no): 2.83, 95% CI: 0.99–8.06, *p* = 0.052 • Awareness of lactation breaks among office workers (yes vs. no): 1.14, 95% CI: 0.33–3.95, *p* = 0.833 • High awareness of policy (office vs. clean room): 2.38, 95% CI: 0.65–8.71, *p* = 0.189 OR for continued breastfeeding after returning to work: • Awareness of lactation room (yes vs. no): 2.71, 95% CI: 1.19–6.15, *p* = 0.017 • Awareness of lactation breaks (yes vs. no): 2.68, 95% CI: 1.57–4.58, *p* < 0.001 • Awareness of lactation room among clean room workers (yes vs. no): 2.06, 95% CI: 0.78–5.40, *p* = 0.143 • Awareness of lactation breaks among clean room workers (yes vs. no): 2.15, 95% CI: 1.09–4.26, *p* = 0.028 • Awareness of lactation room among office workers (yes vs. no): 6.53, 95% CI: 1.33–32.13, *p* = 0.021 • Awareness of lactation breaks among office workers (yes vs. no): 4.91, 95% CI: 1.79–13.46, *p* = 0.002 • High awareness of policy (office vs. clean room): 8.65, 95% CI: 2.48–30.18, *p* = 0.001
Lead Author; Publication Year	Johnston Balkam [34]; 2006
Study Population	Women who had participated in employer’s corporate lactation program within the last 3 years prior to the start of the study and who were still employed by the organization; Maternal race: 69% White, 9% Chinese, 8% Black, 5% Spanish, Hispanic or Latina, 3% Asian Indian, 3% Filipina, 2% Korean, 3.6% other; Maternal education level: 0.8% high school diploma, 10% some college or technical school, 20% Bachelor’s degree, 20% Master’s degree, 48% doctoral degree; Marital status: 97% married, 3% not married; Household income: 30% < USD 100,000, 38% USD 100,000-149,999, 27% ≥ USD 150,000; *N* = 128
Country	USA
Intervention	Prenatal BF classes Telephone support by lactation consultants for new mothers during maternity leave RTW consultation with lactation consultants Ongoing lactation support from lactation consultants during after RTW
Outcomes	Time of program registration and type of feeding at 6 months • Registration before birth: 45% EBF vs. 27% any formula • Registration around birth: 13% EBF vs. 16% any formula • *p* < 0.05 Number of received services and type of feeding at 6 months: • 1 service: 10% EBF vs. 14% any formula • 2 service: 13% EBF vs. 14% any formula • 3 service: 20% EBF vs. 10% any formula • 4 service: 14% EBF vs. 5% any formula • *p* < 0.05
Lead Author; Publication Year	Dabritz et al. [25], 2009
Study Population	Birth mothers who resided in Yolo County, CA at the time of delivery; Maternal age: 15% < 20 years, 25% 20–24 years, 47% 25–34 years, 13% ≥35 years; Maternal ethnicity: 5% Asian, 53% White, 6% others; Maternal education level: 11% ≤8th grade, 15% 9th–11th grade, 73% ≥12th grade; *N* = 399 of which 214 returned to school/work after giving birth
Country	USA
Intervention	Lactation room Lactation break times Workplace/school lactation policy
Outcomes	201 of 214 (94%) infants were at least once breastfed Type of feeding • Presence of lactation room: • Yes: 78% (almost EBF) vs. 68% (partial BF) vs. 64% (no BF) • No: 13% (almost EBF) vs. 28% (partial BF) vs. 26% (no BF) • Did not know: 6% (almost EBF) vs. 4% (partial BF) vs 7% (no BF) • *p* = 0.094 • Lactation break times: • Yes: 92% (almost EBF) vs. 81% (partial BF) vs. 79% (no BF) • No: 2% (almost EBF) vs. 4% (partial BF) vs. 7% (no BF) • Did not know: 6% (almost EBF) vs. 15% (partial BF) vs 14% (no BF) • *p* = 0.22 • Knowledge of lactation policy: • Yes: 79% (almost EBF) vs. 61% (partial BF) vs. 61% (no BF) • No: 21% (almost EBF) vs. 39% (partial BF) vs. 39% (no BF) • *p* = 0.018
Lead Author; Publication Year	Balkam et al. [26]; 2011
Study Population	Female employees working on the employer’s campus, who had finished workplace lactation program within 3 years prior to study and were still employed by the organization in April 2005; Mean maternal age at delivery: 34.4 ± 4.0 years; Marital status: 97% married, 3% not married; Maternal race: 70% White, 30% non-White; Maternal education level: 48% doctoral degree, 20% Master’s degree, 20% Bachelor’s degree, 10% some college or tech school, 2% high school diploma or less; Household income: 31% < USD 100,000, 40% USD 100,000-149,999, 29% ≥ USD 150,000; *N* = 128
Country	USA
Intervention	Prenatal BF education Telephone support RTW consultation Lactation room
Outcomes	EBF 6 months: • Registered for program: 57% • Time of registration: 62.6% before birth vs. 43.2% around RTW; *p* < 0.05 • Prenatal education: 57.4% yes vs. 57.5% no; NS • Telephone support: 62.8% yes vs. 45.2% no; *p* < 0.05 • RTW consultation: 68.0% yes vs. 41.5% no; *p* < 0.05 • Lactation room: 59.8% vs. 48.4% no; NS • # services received: 41.9% 1 service, 47.1% 2 services, 66.6% 3 services, 75.0% 4 services, *p* < 0.05 Any BF 6 months: • Registered for program: 85.9% • Time of registration: 83.5% before birth vs. 91.9% around RTW; NS • Prenatal education: 81.5% yes vs. 90.4% no; NS • Telephone support: 83.7% yes vs. 90.5% no; NS • RTW consultation: 92.0% yes vs. 77.4% no; *p* < 0.05 • Lactation room: 88.7% vs. 77.4% no; NS • # services received: 83.9% 1 service, 85.3% 2 services, 84.6% 3 services, 91.7% 4 services, NS
Lead Author; Publication Year	Weber et al. [52]; 2011
Study Population	Female employees of Sydney South West Area Health Service who took maternity leave between January 2008 and August 2009 with a valid home address; Mean maternal age: 35 years; Maternal background: 66% English speaking, 34% non-English speaking; Marital status: 97% married/de-factor, 1% divorced/separated, 2% never married; Maternal education level: 84% university, 13% technical or trade certificate or diploma, 3% less than tertiary education, 1% other; Household income: 10% < AUD 39,999, 33% AUD 40,000-79,999, 58% ≥ AUD 80,000; *N* = 496
Country	Australia
Intervention	Flexible work practice 30-min paid lactation break per shift (only for women under the nursing and midwifery award)
Outcomes	98% initiated BF Discontinued BF: • 13% at 3 months • 24% at 6 months 59% intended to BF after RTW, 40% BF after RTW How BF and work was combined: • 37% BF before and after work/infant formula during work hours • 36% BF before and after work/expressed BM during work hours • 1% BF before, after and during work hours • 26% other
Lead Author; Publication Year	Bai et al. [33]; 2013
Study Population	Working mothers aged ≥18 years who are currently BF or had BF within 18 months prior to the beginning of the study and who were either staff or faculty at a higher-education institution or who gave birth in the spring and fall of 2010 in one hospital obstetrics unit; Maternal mean age: 33.8 ± 6.0 years; Maternal education level: 2.7% high school, 15.0% some college, 40.7% college graduate, 41.6% postgraduate; Maternal race: 1.8% African American, 2.7% Asian, 2.7% Hispanic, 89.4% White, 3.5% other; Marital status: 92% married, 8% single; *N* = 113
Country	USA
Intervention	N/A
Outcomes	Perceived workplace lactation support and EBF duration: • Technical support: *r* = 0.71, *p* = 0.01 • Workplace environment: r:0.26, *p* = 0.01 • Break time: *r* = 0.05, *p* = 0.52 • Workplace policies: *r* = 0.13, *p* = 0.24
Lead Author; Publication Year	Tsai [42]; 2013
Study Population	Female employees of Company C who have taken maternity leave between January 2009 and January 2011; Maternal age: 23.9% 20–29 year, 74.6% 30–39 years, 1.5% ≥40 years; Maternal education level: 28.3% high school education and below, 71.7% college and above; Worksite: 44.8% clean room, 55.2% office; Shift work: 46.7% yes; Work hours per day: 16.7% 8 h, 83.3% 9–14 h; *N* = 715
Country	Taiwan
Intervention	Lactation facilities (independent space or no independent space, only curtains for separation)
Outcomes	OR for continued BF for 1–6 months after return to work: • Using lactation breaks (yes vs. no): 33.1, 95% CI: 18.0–64.1, *p* < 0.0001 • Supportive co-worker (yes vs. no): 2.53, 95% CI: 2.21–5.32, *p* = 0.0133 • Supportive supervisor (yes vs. no): 2.45, 95% CI: 1.17–5.05, *p* = 0.0156 • NS difference for worksite, shift work, daily working hours, type of lactation room, awareness of lactation breaks OR for continued BF for > 6 months after return to work: • Daily working hours (≤8 vs. 9–14): 2.66, 95% CI: 1.16–6.11, *p* = 0.0206 • Type of lactation room (independent vs. no independent space): 2.38, 95% CI: 1.14–6.32, *p* = 0.0284 • Using lactation breaks (yes vs. no): 51.6, 95% CI: 31.2–121.6, *p* < 0.0001 • Supportive co-worker (yes vs. no): 2.78, 95% CI: 1.14–6.76, *p* = 0.0235 • Supportive supervisor (yes vs. no): 2.44, 95% CI: 1.06–5.61, *p* = 0.0355 • NS difference for worksite, shift work, awareness of lactation breaks
Lead Author; Publication Year	Cohen et al. [29]; 2014
Study Population	Mothers employed at one of the two participating companies and who participated in the onsite corporate lactation program between 1989 and 1992, returned to work for at least 16 h per week after maternity leave; Utilities company (1992): • Average age: 29.5 years (range: 23–41 years) • Average salary: USD 36,000 (range: USD 24,000-50,000) • Average BF duration: 7.4 months (range: 3–14 months) • Maternal race/ethnic origin: 37.9% White, 20.7% African American, 13.8% Asian, 24.1% Hispanic, 3.4% other Aeronautics company (1992): • Average age: 33.1 years (range: 26–40 years) • Average salary: N/A • Average BF duration: 8.4 months (range: 2–16 months) • Maternal race/ethnic origin: 60.0% White, 10.0% African American, 10.0% Asian, 20.0% Hispanic, 0% other *N* = 187
Country	USA
Intervention	Prenatal BF classes Perinatal counseling regarding lactation and RTW lactation maintenance services Provision of electric breast pump
Outcomes	Utilities company (100 birth/year; 1992): • Women returning to work BF and pumping: 29% • Women still in program 6 months after birth: 23% • Women still in program 1 year after birth (1991): 8% Aeronautics company (30 birth/year; 1992): • Women returning to work BF and pumping: 67% • Women still in program 6 months after birth: 47% • Women still in program 1 year after birth (1991): 9.5%
Lead Author; Publication Year	Spatz et al. [27]; 2014
Study Population	Female employees of Children’s Hospital of Philadelphia (CHOP) who filed for maternity leave between 2007 and 2011 and had a CHOP email address; Maternal ethnicity: 75.8% White/Caucasian, 13.0% Black/African American, 7.9% Asian/Pacific Islander, 2.9% Hispanic American, 0.4% American Indian/Alaskan Native; maternal age at delivery (years): 0.2% < 20, 1.8% 20–24, 24.0% 25–29, 47.9% 30–34, 26.1% ≥ 35; *N* = 545
Country	USA
Intervention	Employee lactation policy Personal pump purchase program Pump loaner program from off-campus locations Prenatal BF class BF resource nurse class Lactation rooms
Outcomes	94.5% initiated BF EBF 3 months: 62.9% EBF 6 months: 35.0%
Lead Author; Publication Year	Tsai [43]; 2014
Study Population	Female employees of Company C who have taken maternity leave between January 2009 and January 2011; Maternal age: 23.9% < 30 years, 76.1% ≥30 years; Maternal education level: 28.3% ≤ high school, 71.7% ≥ college; Worksite: 44.8% clean room, 55.2% office; Shift work: 46.7% yes; Work hours per day: 16.7% 8 h, 83.3% 9–14 h; *N* = 715
Country	Taiwan
Intervention	Lactation room with table, chair, sink, electrical outlets and refrigerator 2 lactation breaks of 30 min each per working day
Outcomes	Differences in use of breast-pumping breaks according to age (*p* = 0.0459), maternal education (*p* < 0.0001), husband’s education (*p* = 0.0002), worksite (*p* < 0.0001; clean room uses less often, office uses more often), shift work (*p* < 0.0001; shift workers use less often), NS difference according to work hours per day (*p* = 0.5164) Positive association between the use of lactation breaks after returning to work and awareness about lactation rooms (*p* = 0.0173), breast-pumping breaks policy (*p* < 0.0001), support from supervisor (*p* < 0.0001) and coworkers (*p* < 0.0001), encouragement to use lactation room from environmental health nurses (*p* < 0.0001) and provision of lactation consultant by employer (*p* = 0.0074) Negative association between the use of lactation breaks after returning to work and feeling of embarrassment (*p* = 0.0046), perception of inefficiency (*p* < 0.0001) and believing that lactation breaks would affect supervisor’s assessment of performance (*p* = 0.0079) OR for intention to use breast-pumping break after returning to work: • Awareness of lactation room: 2.27, 95% CI: 0.64–11.00, *p* = 0.2408 • Awareness of lactation breaks: 4.70, 95% CI: 2.90–7.88, *p* < 0.0001 • Provision of lactation consultant: 0.97, 95% CI: 0.67–1.42, *p* = 0.8207 • Guilty feelings when using breast-pumping breaks: 0.81, 95% CI: 0.54–1.21, *p* = 0.3148 • Coworker support: 1.76, 95% CI: 1.01–3.13, *p* = 0.0500 • Supervisor support: 1.47, 95% CI: 0.86–2.51, *p* = 0.1522 • Encouragement by environmental health nurses: 1.16, 95% CI: 0.68–1.95, *p* = 0.5762 • Perception of work inefficiency: 0.55, 95% CI: 0.37–0.82, *p* = 0.0031 • Perception of influenced supervisor’s work evaluation: 1.07, 95% CI: 0.71–1.59, *p* = 0.7470 • Awareness of BF benefits: 1.08, 95% CI: 1.02–1.12, *p* = 0.0050
Lead Author; Publication Year	Basrowi et al. [55]; 2015
Study Population	Female employees of five workplaces in Jakarta whose children were between 6 and 36 months of age and who completed the questionnaire between December 2012 and February 2013; Maternal education level: 24.7% low, 38.7% middle, 36.6% high; Status of home: 36.6% owned, 41.4% rented, 22.0% owned by relatives; *N* = 186
Country	Indonesia
Intervention	Lactation room with refrigerator, hand washing facilities and dedicated seat or bed
Outcomes	OR EBF: • Workplace (office vs. factory): 3.33, 95% CI: 1.77–6.25, *p* < 0.001 • Proper dedicated BF facility (yes vs. no): 2.62, 95% CI: 1.27–5.38, *p* = 0.008 • BF support program (yes vs. no): 5.93, 95% CI: 1.78–19.79, *p* = 0.001
Lead Author; Publication Year	Froh et al. [38]; 2016
Study Population	Female employees of Children’s Hospital of Philadelphia (CHOP) who filed for maternity leave between 2007 and 2011 and had a CHOP email address; *N* = 410
Country	USA
Intervention	• Employee lactation policy • Personal pump purchase program • Pump loaner program from off-campus locations • Prenatal BF class • BF resource nurse class • Lactation rooms
Outcomes	5 major themes • Positive reflections • Non-supportive environment/work culture • Available resources but work culture does not allow to make use of the resources • Perception of non-supportive supervisors and co-workers – missing understanding for situation, understaffed departments, no knowledge about needed pump frequency • Supportive environment/work culture • Environment made the mothers feel comfortable with their BF choice • Accessibility of resources • Not all employees are aware of resources or know how to access them • Difficulties navigating work and BF – busy work schedules, occupied lactation rooms • Internal barriers
Lead Author; Publication Year	Lee et al. [45]; 2015
Study Population	Disproportionate probability sample based on maternal residence in 25 cities/counties in Taiwan: Mothers aged ≥20 years who gave birth in 2008, 2009, 2010 or 2011 and infant was alive at the time of the interview; Maternal age: • 20–24 years: 7.7% (2008), 6.5% (2009), 7.5% (2010), 7.2% (2011) • 25–29 years: 28.4% (2008), 26.9% (2009), 28.4% (2010), 26.9% (2011) • 30–34 years: 40.9% (2008), 42.7% (2009), 42.8% (2010), 43.1% (2011) • ≥35 years: 23.0% (2008), 23.9% (2009), 21.3% (2010), 22.9% (2011) Maternal education level: • ≤ junior high school: 12.2% (2008), 8.3% (2009), 7.0% (2010), 6.0% (2011) • High school: 38.2% (2008), 31.6% (2009), 29.7% (2010), 29.1% (2011) • Vocational school: 26.4% (2008), 26.6% (2009), 21.8% (2010), 21.6% (2011) • ≥ university: 23.3% (2008), 33.5% (2009), 41.5% (2010), 43.3% (2011) Employed outside the home: 65.0% (2008), 69.6% (2009), 57.0% (2010), 55.5% (2011); *N* = 2163 (2008); 1453 (2009); 11,011 (2010); 12,410 (2011)
Country	Taiwan
Intervention	Lactation room
Outcomes	OR of EBF: 2.68, 95% CI: 2.44–2.94, *p* < 0.001 OR of any BF: 3.25, 95% CI: 2.99–3.53, *p* < 0.001
Lead Author; Publication Year	Kozhimannil et al. [28]; 2016
Study Population	Women who gave birth in U.S. hospital between July 2011 and June 2012 and who were employed at the time of the follow-up survey between January and April 2013; Maternal age: 27.5% 18–24 years, 25.9% 25–29 years, 26.8% 30–34 years, 19.7% ≥35 years; Maternal race: 62.3% White, 13.7% Black, 18.0% Hispanic, 6.1% other/multiple race; Maternal education level: 26.3% ≤ high school, 28.4% some college/associate’s degree, 27.3% Bachelor’s degree, 18.0% graduate education/degree; Income: 32.3% ≤ USD 52,300, 47.4% USD 52,301-102,000, 20.3% > USD 102,001; *N* = 550
Country	USA
Intervention	Lactation break times Private lactation space
Outcomes	PP employment plans affected BF-related decision • Sufficient break time (yes): 46.9%, *p* = 0.302 • Private room (yes): 46.7%, *p* = 0.379 • Break time + private room (yes): 44.0%, *p* = 0.122 Employment posed a challenge to BF • Sufficient break time (yes): 35.2%, *p* = 0.371 • Private room (yes): 33.3%, *p* = 0.918 • Break time + private room (yes): 31.0%, *p* = 0.570 BF intention at the end of pregnancy • Sufficient break time (yes): *p* = 0.17 • BF only: 62.6% • Formula only: 10.7% • BF + formula: 26.7% • Private room (yes): *p* = 0.163 • BF only: 57.7% • Formula only: 11.3% • BF + formula: 31.1% • Break time + private room (yes): *p* = 0.189 • BF only: 59.7% • Formula only: 10.6% • BF + formula: 29.7% BF status at 6 months • Sufficient break time (yes): *p* = 0.030 • BF only: 71.4% • Formula only: 14.7% • BF + formula: 14.0% • Private room (yes): *p* = 0.677 • BF only: 75.7% • Formula only: 11.9% • BF + formula: 12.4% • Break time + private room (yes): *p* = 0.722 • BF only: 75.1% • Formula only: 12.4% • BF + formula: 12.5% Mean EBF duration (months) • Sufficient break time (yes): 5.37, *p* = 0.397 • Private room (yes): 5.89, *p* = 0.002 • Break time + private room (yes): 5.64, *p* = 0.088 AOR of EBF at 6 months • Reasonable break time to express milk: 2.593, 95% CI: 1.00–6.71 • Private room to express milk: 2.669, 95% CI: 0.43–16.48 • Break time + private room: 2.255, 95% CI: 1.03–4.95 AOR of any BF at 6 months • Reasonable break time to express milk: 3.004, 95% CI: 1.23–7.32 • Private room to express milk: 0.555, 95% CI: 0.12–2.57 • Break time + private room: 1.946, 95% CI: 0.88–4.28
Lead Author; Publication Year	Paddock [24]; 2017
Study Population	Cornell employees with at least one dependent child aged 12 years or younger in February 2009; Maternal education level: 47.7% graduate degree, 34.3% college degree, 12.2% attended college, 5.6% completed high school; *N* = 919
Country	USA
Intervention	Financial support to spend on any legal childcare for children up to age 13 years
Outcomes	BF was associated with higher education, marriage, higher income, academic position bs. Hourly position and work unit Inflexible work schedule as reason for not initiating/stopping BF Missing information about BF rights as reason for not initiating/stopping BF Flexible work schedule/availability to work part-time as BF facilitator
Lead Author; Publication Year	Butudom [50], 2018; quantitative analysis
Study Population	Mothers working for Company A and who took maternity leave between June and December 2016; Maternal age: 5.6% < 25 years, 38.4% 25–29 years, 32.4% 30–34 years, 20.4% 35–39 years, 3.2% ≥40 years; Maternal education level: 15.3% < high school, 56.9% high school, 21.8% vocational degree, 6.0% ≥ Bachelor’s degree; Marital status: 10.6% single, 2.3% separated/divorced, 87.0% married; Monthly income: 10.6% < Baht 10,000, 87.0% Baht 10,000-19,999, 0.9% Baht 20,000-29,999, 1.4% ≥ Baht 30,000; *N* = 216
Country	Thailand
Intervention	Fully equipped lactation room Breast pumping equipment Refrigerator to store expressed BM BM drop-off service BF training program
Outcomes	EBF rate: 76.9% at 1 month, 46.3% at 3 months, 7.4% at 6 months Reported reasons to stop BF: 36% insufficient milk supply, 31% infant lives with grandmother too far away, 12% RTW, 21% other reasons
Lead Author; Publication Year	Butudom [50], 2018; qualitative analysis
Study Population	Mothers working for Company A and who took maternity leave between June and December 2016; Mean maternal age: 31.70 ± 4.46 years; Monthly income: 16.7% < Baht 10,000, 73.3% Baht 10,000-19,999, 10.0% ≥ Baht 20,000; *N* = 30
Country	Thailand
Intervention	Fully equipped lactation room Breast pumping equipment Refrigerator to store expressed BM BM drop-off service BF training program
Outcomes	BF policy was seen as helpful to decide to BF the infant Impact of RTW on BF • Infant living in distant location: grandmothers are lacking knowledge how to feed frozen BM; mothers are concerned that expressed/frozen BM will be spoiled because of the long transport (2–3 days) • BM transportation: difficult to send expressed/frozen BM because of inadequate public transport • Importance of social support during BF
Lead Author; Publication Year	Payton [35]; 2018; quantitative analysis
Study Population	Women BF a biological child within the last 5 years while being full-time employed by organization who is member of the Greater Philadelphia Business Coalition on Health or the Pittsburgh Business Group on Health; Maternal mean age: 33.65 ± 4.07 years; Maternal race: 7% Asian, 11% Black or African American, 76% White, 4% mixed, 3% other; Maternal education level: 2% high school, 12% some college, 45% college degree, 42% graduate degree, 1% not documented; Marital status: 22% married/with partner, 1% not married/with partner, 78% not documented; *N* = 199
Country	USA
Intervention	N/A
Outcomes	64% BF as long as intended BF durations and achieved BF goals • 77% of mothers who BF for > 6 months, BF for as long as intended • 51% of mothers who BF for 3–6 months, BF for as long as intended • 37% of mothers who BF between 6 weeks and 3 months, BF for as long as intended • 25% of mothers who BF for < 6 weeks, BF for as long as intended • *p* < 0.001 BF location at workplace and achieved BF goals • 58% of mothers who pumped in a bathroom did not BF for as long as intended • 29% of mothers who did not pump in a bathroom BF for as long as intended • *p* < 0.001 Significant association between BF intention and perceptions of workplace lactation support with BF duration, *p* < 0.001 Significant association between perception of workplace lactation support BF duration for ≥6 months, *p* < 0.001 Significant association between utilization of workplace lactation support and BF duration for ≥6 months, *p* < 0.001
Lead Author; Publication Year	Payton [35]; 2018; qualitative analysis
Study Population	Women BF a biological child within the last 5 years while being full-time employed by organization who is member of the Greater Philadelphia Business Coalition on Health or the Pittsburgh Business Group on Health; Maternal mean age: 32.86 ± 3.72 years; Maternal race: 14% Black or African American, 64% White, 21% other/mixed; Maternal education level: 21% some college, 17% college degree, 58% graduate degree; Marital status: 93% married/with partner, 7% not married/with partner; *N* = 14
Country	USA
Intervention	N/A
Outcomes	Main themes from interviews with BF employees • Cognitive influences on behavior • Environmental influences on behavior • Supporting behavioral factors
Lead Author; Publication Year	Santos et al. [47]; 2018
Study Population	Mothers working at a higher education institution but not being a student or resident at the time of the birth of the infant as well as at the time of the study; infants attending childcare center at the mother’s workplace; Maternal age: 47.8% < 35 years, 52.2% ≥35 years; Maternal education level: 65.2% higher education, 26.1% vocational education, 8.7% medium; *N* = 46
Country	Brazil
Intervention	Child-care center Lactation breaks (2 × 30-min breaks or 1-h reduction in workload)
Outcomes	Median BF duration according to availability of lactation breaks • Lactation breaks: 120 days (25 percentiles: 90 days – 75 percentiles: 180 days) • No lactation breaks: 150 days (25 percentiles: 120 days – 75 percentiles: 150 days) • *p* = 0.5148
Lead Author; Publication Year	Wambach et al. [30]; 2018
Study Population	Registered nurses who have been employed at the hospital for a minimum of 2 years and concurrently BF or had done so within the past 12 months; Maternal education level: 11.5% associate’s degree, 78.2% Bachelor’s degree, 10.3% Master’s degree; Maternal race: 1.3% Asian, 98.7% White; Maternal ethnicity: 1.3% Hispanic or Latino, 98.7% non-Hispanic or non-Latino; *N* = 78
Country	USA
Intervention	N/A
Outcomes	94% had designated lactation space Positive correlation between Workplace Breastfeeding Support Scale (WBSS) subscale “Break Time” and BF duration: *r* = 0.335, *p* = 0.035 NS correlation between WBSS subscales “Environment”, “Technical Support”, “Workplace Policy” and BF duration
Lead Author; Publication Year	Chen et al. [53]; 2019; quantitative analysis
Study Population	Mothers with children under 12 months who are living in one of the 12 randomly chosen county level regions (4 urban cities, 4 small and medium sized cities, 2 rural areas, 2 poor rural areas); Mean maternal age: 29.15 ± 5.11 years; Maternal education level: 8.08% ≤ primary school, 36.53% middle school, 18.04% high/vocational school, 37.35% ≥ college; Employment status: 69.66% informal, 30.34% formal; *N* = 9725
Country	China
Intervention	N/A
Outcomes	AOR of early BF initiation (unemployment as base) • Agriculture related occupation: 1.32, 95% CI: 1.15–1.51 • Industry related occupation: 1.00, 95% CI: 0.75–1.34 • Business and white-collar occupation: 1.38, 95% CI: 1.23–1.56 AOR of EBF for 0–6 months (unemployment as base) • Agriculture related occupation: 1.30, 95% CI: 1.04–1.62 • Industry related occupation: 0.77, 95% CI: 0.44–1.34 • Business and white-collar occupation: 0.95, 95% CI: 0.78–1.17
Lead Author; Publication Year	Chen et al. [53]; 2019; qualitative analysis
Study Population	Mothers with children under 12 months who are living in one of the 12 randomly chosen county level regions (4 urban cities, 4 small and medium sized cities, 2 rural areas, 2 poor rural areas); Maternal education level: 6% primary school, 27% middle school, 20% high school, 47% ≥ college; Household income: 27% ≤ yuan 50,000, 35% yuan 50,000-100,000, 24% yuan 100,000-200,000, 14% ≥ yuan 200,000; *N* = 84
Country	China
Intervention	Lactation breaks
Outcomes	Themes from interviews 1) Employment benefits: Formal employment can provide maternal benefits ensured by law and regulations • Paid maternity leave: Mothers wish to extend paid maternity leave (min. 98 days) to be able to adhere to WHO BF recommendations, provision of longer unpaid breaks often difficult because of financial needs of families • BF breaks: Mothers feel encouraged by BF breaks (1 h per workday for infant < 1 year) to continue BF after RTW. But if commute time is too long, mothers do not feel able to continue to BF after RTW despite provision of BF breaks 2) Commute time: Length of commute time determines if formally employed mothers feel able to continue BF after RTW; for informally employed mothers, length of commute time determines if family support was accessible 3) Workplace environment: Use of electric breast pumps as alternative for direct BF among working mothers, but concerns about physical lactation environment at the workplace • Space for Lactation: Social support as well as private and clean space as necessity to continue BF • Equipment for pumping: limited equipment (possibilities to store BM, power outlets for breast pumps) at workplace as challenge to continue BF 4) Labor intensity: Work schedule and workload influences frequency of BF or use of breast pump • Flexibility of work schedule: Flexible work schedule as supporting factor; night shifts and irregular work schedules as reason for weaning • Stress from work: BF with busy work schedule was tiring; high level of stress as a reason for perceived decrease of BM supply
Lead Author; Publication Year	Scott et al. [31]; 2019
Study Population	Female adult employees (age ≥ 18 years) of the health care system who have been employed for ≥6 months and BF in the past 3 years prior to the study; Maternal age: 72.7% ≤35 years, 27.3% > 35 years; Maternal race: 76.8% White, 16.2% Black, 7.0% other; Marital status: 88.8% married, 8.4% never married, 2.8% other; Maternal education level: 26.5% ≤ some college, 39.2% college degree, 34.3% graduate degree; *N* = 165
Country	USA
Intervention	N/A
Outcomes	OR of BF duration • Organizational support: 1.05, 95% CI: 0.84–1.32, *p* = 0.65 • Managerial support: 1.13, 95% CI: 0.88–1.44, *p* = 0.34 • Co-worker support: 0.88, 95% CI: 0.67–1.14, *p* = 0.32 OR of EBF • Organizational support: 1.81, 95% CI: 1.06–3.09, *p* = 0.03 • Managerial support: 0.87, 95% CI: 0.50–1.49, *p* = 0.61 • Co-worker support: 0.89, 95% CI: 0.52–1.54, *p* = 0.69 OR of EBF duration • Organizational support: 1.10, 95% CI: 0.76–1.60, *p* = 0.61 • Managerial support: 1.47, 95% CI: 1.03–2.09, *p* = 0.03 • Co-worker support: 0.83, 95% CI: 0.62–1.12, *p* = 0.22
Lead Author; Publication Year	Ibarra-Ortega et al. [56]; 2020
Study Population	Mothers working at institutions with more than 251 employees in Guadalajara, Mexico, with children aged between 6 and 35 months and who were working when BF was initiated; Maternal mean age: 34.9 ± 4.3 years (with lactation room) vs. 31.4 ± 4.7 years (no lactation room); Maternal education level: 21.1% ≤ high school, 78.9% ≥ college vs. 26.8% ≤ high school, 73.2% ≥ college; Marital status: 17.1% single, 78.9% married, 4.0% others vs. 36.6% single, 58.5% married, 4.9% other; *N* = 158
Country	Mexico
Intervention	Lactation room
Outcomes	OR of BF duration ≥6 months (lactation room vs. no lactation room): 3.15, 95% CI: 1.60–6.19, *p* = 0.001 OR of BF duration ≥12 months (lactation room vs. no lactation room): 2.69, 95% CI: 1.23–5.86, *p* = 0.014 OR of EBF duration ≥6 months (lactation room vs. no lactation room): 2.53, 95% CI: 1.16–5.54, *p* = 0.022 OR of EBF at 6th months (vs. partial BF) (lactation room vs. no lactation room): 2.98, 95% CI: 1.41–6.29, *p* = 0.006 NS difference in EBF and BF duration between mother who had access to lactation room but did not use lactation room and mothers without lactation room
Lead Author; Publication Year	Kebede et al. [54]; 2020
Study Population	Permanently employed mothers with children aged 6–24 months working in governmental and/or nongovernmental organization in Dukem town; Mean maternal age: 27.1 ± 3.44 years; Maternal age: 16.6% 18–23 years, 60.4% 24–29 years, 23.0% ≥30 years; Maternal ethnicity: 90% Oromo, 1.0% Tigre, 6.7% Amara, 1.9% others; Maternal education level: 19.8% secondary, 44.1% diploma, 36.1% ≥ degree; Marital status: 1.3% single, 92.7% married, 5.4% divorced, 0.6% widowed; Income: 1.3% ≤ Ethiopian Birr 500, 15.3% Ethiopian Birr 501–1000, 8.9% Ethiopian Birr 1001–1500, 16.9% Ethiopian Birr 1501–2000, 57.5% > Ethiopian Birr 2001; *N* = 313
Country	Ethiopia
Intervention	Lactation break
Outcomes	OR of EBF discontinuation • No lactation break: 6.7, 95% CI: 3.0–14.5 • BF at workplace: 3.5, 95% CI: 1.7–7.2 • Pumping BM: 4.3, 95% CI: 1.7–11.0

*95% CI* 95% confidence interval, *AOR* adjusted odds ratio, *BF* breastfeeding, *BM* breastmilk, *EBF* exclusive breastfeeding, *N/A* not applicable, *NS* not significant, *OR* odds ratio, *RTW* return to work

**Table 7:** Summary of Publications Included in the Realist Analysis Following a Posttest Design. Realist Review on “How Do Breastfeeding Workplace Interventions Work?”

POSTTEST DESIGN	
Lead Author; Publication Year	Dodgson et al. [36]; 1997
Study Population	Women who studied or worked at University of Minnesota and used the lactation room during the first 18 months of its existence; RTW: 36 yes (15 students, 15 staff, 6 faculty) – 10 no (7 students, 3 staff); *N* = 46
Country	USA
Intervention	Lactation room Orientation package incl. Information about pump use, community lactation resources, collection and storage of BM Individual consultation and educational program Information and educational material related to BF for the lactation room and departments upon request Telephone BF consultations
Outcomes	Perceived impact on BF (1 being the least positive response, 7 being the most positive response; mean ± SD) • Increased use of BM instead of formula: 6.02 ± 1.57 • Increased length of BF duration: 5.27 ± 2.18 EBF rates among mothers who returned • 1 months: 91.4% • 3 months: 80.6% • 6 months: 47.2%

*BF* breastfeeding, *BM* breastmilk, *RTW* return to work, *SD* standard deviation

**Table 8:** Summary of Publications Included in the Realist Analysis Following a Pretest-Posttest Design. Realist Review on “How Do Breastfeeding Workplace Interventions Work?”

PRETEST-POSTTEST DESIGN	
Lead Author; Publication Year	Katcher et al. [37]; 1985
Study Population	Female employees of Department of Pediatrics at Hunterdon Medical Center, New Jersey; Pretest group (*N* = 19): Mothers who took maternity leave between September 12, 1979 and May 27, 1981, before support program was implemented; Posttest group (*N* = 22): Mothers who returned to work between July 2, 1981 and January 7, 1983, after support program was implemented
Country	USA
Intervention	Lactation room Electric breast pump (stored in the office of the Employee Health Service) Assistance by Employee Health Service (pump instruction, access to lactation room, information about BM storage and use at home) Refrigerator to store expressed BM BF counseling
Outcomes	BF initiation: 16 out of 19 (pretest) vs. 22 out of 22 (posttest) Discontinuation of BF before RTW: 7 out of 16 (pretest) vs. 0 out of 22 (posttest), *p* < 0.003 Average EBF duration (weeks): 10.6 (pretest) vs. 12.1 (posttest), *p* < 0.003 Average total BF duration (months): 6.0 (pretest) vs. 11.7 (posttest), *p* < 0.003
Lead Author; Publication Year	Rea et al. [48]; 1997
Study Population	Women working in factories in São Paulo (70% blue collar workers) who are in their third pregnancy trimester; Interview in third pregnancy trimester (*N* = 76); Re-interview after RTW (*N* = 69)
Country	Brazil
Intervention	Childcare at worksite Lactation room for BM extraction and BM storage Schedule flexibility Not working in production line
Outcomes	Factors associated with longer BF duration: • Higher socioeconomic status • Childcare at worksite • Lactation room for BM extraction and BM storage • Not working during weekend • Not working in production line
Lead Author; Publication Year	Yimyam et al. [51]; 2014
Study Population	Employed mothers; Pretest group: *N* = 24; Posttest group: *N* = 33
Country	Thailand
Intervention	BF education by nurse-midwives and/or lactation consultants in cooperation with nurses at the workplace BF support by nurse-midwives and/or lactation consultants in cooperation with nurses at the workplace Lactation room BF support campaigns at the workplace
Outcomes	EBF rate at 6 months: 4.2% (pretest) vs. 36.4% (posttest), *p* = 0.004 Any BF rate at 6 months: 29.2% (pretest) vs. 57.6% (posttest), *p* = 0.033

*BF* breastfeeding, *BM* breastmilk, *EBF* exclusive breastfeeding, *RTW* return to work

**Table 9:** Summary of Publications Included in the Realist Analysis Following a Qualitative Analysis. Realist Review on “How Do Breastfeeding Workplace Interventions Work?”

QUALITATIVE ANALYSIS	
Lead Author; Publication Year	Osis et al. [49]; 2004
Study Population	Women working at a public university with access to the institution’s childcare program; Focus group discussion; 15 women EBF their babies, 15 women whose babies were already being fed with other food besides BM; of these 30 women, 20 agreed to participate in 2 focus group discussions (10 women per focus group discussion)
Country	Brazil
Intervention	Childcare at workplace
Outcomes	Free childcare at the workplace may facilitate EBF once women return to work
Lead Author; Publication Year	Hilliard [23], Chapter 6; 2018
Study Population	Working women in North Dakota who gave birth to a child between 2014 and 2016 and who attempted to continue BF after RTW; Predominantly white (97%), married (94%) participants holding a bachelor’s degree or higher (77%); *N* = 392
Country	USA
Intervention	N/A
Outcomes	Positive association for BF duration: • Maternal self-efficacy for BF and BF duration, *p* = 0.01 • Maternal self-efficacy for combining work and BF and BF duration, *p* = 0.00 • Maternal comfort to ask for lactation accommodation, *p* = 0.00 • Maternal perception of supportive co-worker, *p* = 0.00 • Maternal comfort to take lactation breaks, *p* = 0.00 • Maternal comfort to adjust break schedule to meet pumping needs, *p* = 0.00 Negative association for BF duration: • Maternal perception that number of hours worked made it difficult to combing BF and working, *p* = 0.00 • Maternal perception of insecure job, *p* = 0.04 NS association for BF duration: Maternal perception of supportive manager, *p* = 0.75

*BF* breastfeeding, *BM* breastmilk, *EBF* exclusive breastfeeding, *N/A* not applicable, *NS* not significant, *RTW* return to work

The intervention sample in all but one study, was conformed by employed women. Sahip and Turan [58] described the effects of a workplace health program for expectant fathers in Turkey. The intervention was delivered by specially trained workplace physicians and consisted of six 3–4 h sections: 1) health during pregnancy, 2a) pregnancy nutrition, 2b) birth, 3) communication techniques, 4) infant health care and feeding, 5) fatherhood, and 6) family health after birth. Children of fathers in the education group had 2.38 times the odds to be breastfed within the first hour after birth than children of fathers in the control group (95% confidence interval (CI): 1.24–4.61). Also, children of fathers in the education group had 3.44 (95% CI: 1.74–6.82) times the odds for exclusive breastfeeding at 3 months, 2.64 (95% CI: 1.36–5.09) times the odds for any breastfeeding at 9 months and 0.19 (95% CI: 0.09–0.37) times the odds for supplementary feeding before 6 months compared to children of fathers in the control group.

### Realist analysis

**Fig. 2:**
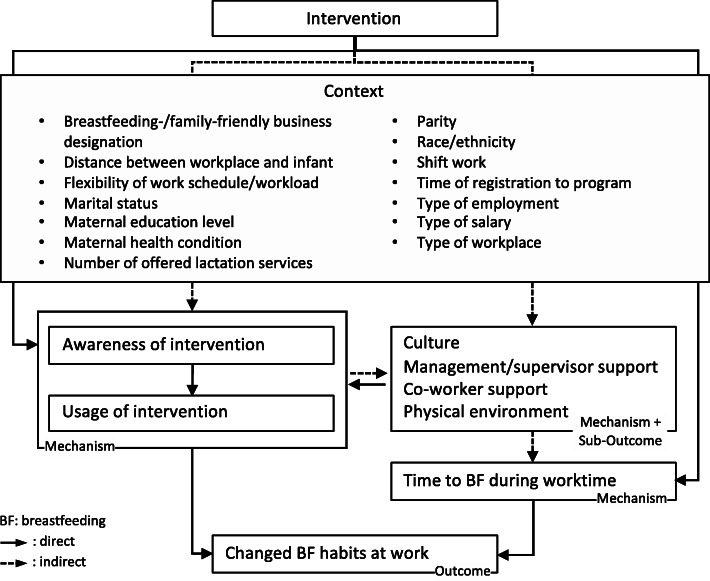
Context-Mechanism-Outcome Framework of Breastfeeding Interventions at the Workplace. Realist Review on “How Do Breastfeeding Workplace Interventions Work?”

To better understand how workplace breastfeeding interventions work and how their influence on breastfeeding outcomes differs in different contexts, we categorized the interventions described in the analyzed articles into 4 types of intervention and 15 intervention categories (Table 10). To develop a CMO framework (Fig. 2), we analyzed the outcomes per intervention category. We concentrated our analysis on the outcomes on changed breastfeeding behavior as well as changes in perceived workplace breastfeeding culture (e.g., how common breastfeeding at the workplace is), manager/supervisor support, co-worker support and in the physical environment. It is to mention, that workplace breastfeeding outcomes can also lead to additional outcomes that were out of scope of this review such as job satisfaction [22, 31].

**Table 10:** Intervention Types and Categories. Realist Review on “How Do Breastfeeding Workplace Interventions Work?”

Intervention type	Intervention category
Provision of equipment/facility	Lactation room
	Electric breast pump
	Refrigerator
	Day-care center
Provision of time	Lactation break times
	Allowance of flexible break times/work schedule
Education	Breast pump instruction
	Prenatal breastfeeding education
	Breastfeeding education, time not specified
	Lactation counseling
	Return to workplace counseling
	Expectant father education
	Provision of breastfeeding information (written or verbally)
Human resource policy and communication	Workplace lactation policy
	Communication strategy

Our analysis revealed three potential mechanisms or factors that might explain how workplace interventions influence breastfeeding habits at the workplace: 1) having more time to breastfeed during work, 2) awareness of the intervention among mothers, supervisors and co-workers and 3) change in culture, management/supervisor support, co-worker support and/or physical environment. Having more time to breastfeed can be directly achieved for example by providing lactation breaks or flexible working hours or indirectly for example by changing the physical environment (e.g., providing lactation rooms [43, 54]) or co-worker support (e.g., by raising the understanding of co-worker through a communication strategy such that the co-worker support flexible work schedule). The mechanism of awareness about the intervention acts directly on the mother as well as indirectly through raising awareness of others; e.g. supervisors and co-workers who can then direct the mother to the lactation program. As long as the mother is not aware of the intervention, she cannot use it, and thus, her breastfeeding habit at the workplace will not change due to the intervention [25, 43, 44]. Supervisor awareness was also important in order to be able to advise their pregnant employees of corporate lactation programs. Therefore, changes in culture, management/supervisor support, co-worker support and/or physical environment are mechanisms and associated with changes in breastfeeding habits of employed mothers [23, 31, 33, 35, 36, 38, 41, 42].

The analysis of outcomes based on their intervention category also led to the identification of important contextual factors. The context in which the intervention is implemented determines whether it is sufficient to affect breastfeeding outcomes. We identified the following contexts: breastfeeding−/family-friendly business designations [32, 41], distance between workplace and infant [50, 53, 54], flexibility of work schedule/workload [38], marital status [24], maternal education level [24, 31, 57], maternal health conditions [38], number of offered lactation services [26, 28, 34], parity [57], race/ethnicity [24, 26], shift work [29, 40, 42–44], time of registration to program [26, 34], type of employment (full-time vs. part-time) [26], type of salary (fixed vs. hourly) [24, 39], type of workplace [24, 42–44]. As an example of how different work-related contexts require different implementation mechanisms, or, lead to different outcomes for similar interventions, we will concentrate on the context of distance between workplace and infant as well as the contexts of flexibility of work schedule/workload, shift work and type of workplace.

The sole implementation of a workplace breastfeeding intervention was not always sufficient to cause a change in breastfeeding habits at the workplace. We found that a large distance between the workplace and the location of the infant as well as commute times are a major barrier for continued breastfeeding after returning to work. Mothers whose children are living far apart from them described the large distance between them and their infants as a reason to stop breastfeeding after return to work; despite the presence of lactation break policies [50, 53], lactation facilities [50] or even the offer of a drop-off service that brings expressed breastmilk to the local bus and van station from where the breastmilk is transported to rural areas, where the infant is living [50]. Underlying reasons seem to be different: Mothers in Thailand report that they fear that their breastmilk will be spoiled during the long transportation and that grandmothers, who are caring for the infant, lack the knowledge how to handle frozen/expressed breastmilk [50], in China the legal provision of one-hour feeding break for working mothers of infants aged younger than 1 year is insufficient for mothers whose children are not near the workplace since the provision of lactation facilities is not legally required, thus, requiring mothers to travel to their infant to feed them [53]. While the example in Thailand shows that mechanisms outside the work environment are influential, the example in China shows that the sole provision of time can be insufficient if the physical environment does not support breastfeeding.

Other contexts in which the sole implementation of breastfeeding interventions at the workplace are insufficient, are workplaces with a busy and/or inflexible work schedule, workplaces other than an office, and type of work such as shift work. Professions such as nurses or physicians have a work schedule that is often influenced by external factors which contributes to inconsistent scheduling. In such a context, the sole provisions of lactation breaks and lactation facilities are inefficient if mothers perceive their co-workers and supervisors as not supportive of them taking breastfeeding breaks [38]. Inflexible environments (e.g., fabric/production workplaces) and workplaces without a specific office as well as shift work are also contexts in which the sole provision of lactation breaks and lactation facilities are insufficient. Mothers working in a clean room (a room that is maintained free of contaminants) in two Taiwanese manufacturing companies had lower breastfeeding rates than their female colleagues having an office space in the same company [42–44]. Tsai and colleagues found that office workers and non-shift workers used the lactation breaks and facilities available to them more often than workers of the clean room and shift workers. To be able to use the offered lactation breaks, clean room workers needed to fully change from their clean room suits into normal clothes and then dress back into their clean room suits. This may have left less time to pump, thus, making the available lactation break times too short and less effective for breastfeeding. Chen et al. found that among women who were aware of lactation breaks and facilities, office workers had showed a higher likelihood for continued breastfeeding after returning to work than clean room workers indicating that in this context, the awareness mechanism was not mediating the breastfeeding outcomes. Given the rigid work schedule of clean room and shift workers, it is more likely that among them the intervention of breastfeeding breaks and facilities may not increase the time available for breastmilk expression.

Performance bonuses for manufacturing workers may inhibit the activation of support from co-workers, and thus, the activation of the time-releasing mechanism needed for breastfeeding and expressing breastmilk. Performance bonuses for most Taiwanese manufacturer workers are based on group productivity and individual performance [44], thus, breastfeeding mothers working in the clean room dependent on their co-workers to take over their duties while they are breastfeeding in order to keep their own performance bonuses and the ones of their co-workers, which may be unrealistic. Therefore, in order to increase breastfeeding rates among manufacturing and shift workers, workplace lactation interventions need to involve the network of co-workers to indirectly enable breastfeeding workers to use lactation breaks and facilities.

## Discussion

To our knowledge, this is the first realist review identifying potential mechanisms underlying the impact of workplace breastfeeding interventions as well as the effect of different contexts on the influence on breastfeeding outcomes of such interventions. Using a realist review approach, we were able to integrate findings from a plethora of study designs including qualitative studies. An innovative aspect from our review is that we were able to develop a pragmatic context-mechanism-outcome (CMO) framework. This framework shows that contextual factors such as long distances between the workplace and the infant will hinder the influence of the intervention at improving breastfeeding outcomes among working mothers. Specifically, our CMO framework identified three mechanisms at work that need to be activated for an intervention to be effective: 1) the awareness of workers, supervisors and co-workers about the availability of entitlement to a given intervention, 2) changes in: perceived breastfeeding culture at the workplace, including manager/supervisor and co-workers support and adequate physical environments, and 3) having time to breastfeed or express breastmilk during work time.

Positive associations between workplace lactation support and interventions have been shown previously [11, 14–17, 19]. However, systematically understanding how such interventions work has been a major gap. Furthermore, examining how contexts mediate or moderate the influence of workplace breastfeeding interventions across different workforce groups had not been previously done, as far as we know. Our review is impactful because it provides this information at a time when an increasing number of women participate in the labor force [1, 2]. Our review indeed identified mechanisms that if properly taken into account when designing interventions may empower mothers to not have to decide between choosing the best nutrition for their infant or working, thus, helping close a major inequity gap affecting working women with infants globally.

While it is important to lessen breastfeeding inequalities between working and non-working mothers, it is as important to lessen these inequalities among working women. In the course of our analysis, we found that the studies represented a wide spectrum of maternal demographics including age, education level, race and ethnicity, income level, and marital status. Overall, higher breastfeeding rates and longer breastfeeding duration in the workplace were associated with higher maternal education [24, 25, 28, 31, 42–44, 54, 55], higher income levels [25, 28], being White [24–26] and being married or living with a partner [24, 28]. The included studies did not allow us to examine the underlying pathways that may have explained differences in implementation approaches as a result of differences in socioeconomic and demographic contexts because awareness and/or uptake of the interventions were seldomly examined as a function of the afore mention characteristics. Therefore, future research is needed to elucidate how best to tailor work-based breastfeeding interventions to different contexts.

The available data did also not allow us to determine underlying pathways which may explain differences in breastfeeding outcomes of work-based intervention as a function of type of employment (full-time vs. part-time employment). This is because the study authors did not examine awareness and/or uptake of the intervention as a function of the employment status which would be needed to be able to determine differences in awareness and/or uptake of the intervention among women with different employment status as pathway for the differences seen in breastfeeding outcomes among women with different employment status. One study showed a significant association between part-time employment and higher breastfeeding rates at 6 months while two other studies did not find significant associations [34, 39]. Possible explanations of these inconsistent findings are the heterogeneous use of the term “part-time” and the heterogenous group of part-time working mothers. For example, it is clear that the needs for breastfeeding support of a mother working 8 h a week are likely to be different from the needs of a mother working 40 h a week. However, it is unclear how the needs of a mother working 40 h a week would compare with the needs of a mother working 34 h a week, either as a four-day working week or distributed across all weekdays. Moving forward, instead of using unclear terms like “part-time” employment, future research should consider evaluating workplace breastfeeding interventions based on actual hours worked per day and days worked per week.

While this realist review integrated evidence from a plethora of study designs allowing for the examination of how workplace breastfeeding interventions work across various contexts and uncovering potential pathways for impact, it is not without limitations. Firstly, the review did not include studies that were solely focusing on maternity leave benefits. Rather, it focused on lactation interventions for mothers returning to the workplace. We made this decision because previous work has documented the positive impact of extended duration of maternity leave on breastfeeding outcomes [59, 60] and maternity leave policies are beyond the sole domain of the employers. Secondly, in order to bring focus to the review, we limited the search to studies reporting on breastfeeding outcomes. This limitation omitted publications reporting on other outcomes of workplace breastfeeding interventions such as job satisfaction or health costs for employers, employees and/or society. And lastly, as recommended for realist reviews, we used a purposive rather than a comprehensive screening strategy. Thus, it is possible that a relevant paper could have been available in the databases we searched, but was not retrieved by our queries, as we screened only those papers where the workplace context was explicit in either the title and author keywords or subject headings. To mitigate this risk, we searched in multiple subject-indexed databases, on the reasoning that a paper which was poorly indexed in Medline may have been better indexed in CINAHL or Global Health.

Because of time limitations, we did not conduct grey literature searches as well as citation chaining. It is possible that these omissions led to the introduction of biases. Nevertheless, we think that this is unlikely because the vast majority of grey literature about workplace breastfeeding interventions are technical guidelines on how to implement specific interventions such as lactation rooms [61], and do not report on breastfeeding outcomes, thus would not have passed the eligibility criteria for inclusion of the present review. Since we searched a plethora of databases, we are confident that our search picked up the vast majority of eligible publication, thus, we estimate the risk of bias introduction due to the missing citation chaining as minimal.

Our review strongly calls for more mixed methods work-based breastfeeding intervention research in low- and middle-income countries, that also includes the very large number of women working in the informal economy. Of the 37 included studies, only one study was conducted in a low-income country [54] as defined by the World Bank [62]. This is unfortunate, as out of the approximately 7.7 billion people in the world in 2019, 6.5 billion people lived in low-and middle-income countries, and 670 millions lived in low-income countries [63]. None of the included studies of this review focused solely on informal employment and the majority included only formally employed women. While formally employed mothers can be protected by laws and regulations, such as mandated maternity leave, informally employed women may need to depend on other mechanisms that support their informed decisions about infant feeding [53]. Therefore, there is a profound inequity in the selection of settings where the work-based breastfeeding research has been conducted, as well as the type of employment included in those studies (formal vs. informal economy). This is unacceptable given the very high proportion of women employed in the informal sector in low- and middle-income countries [64]. How the policy design and program implementation mechanisms need to differ for delivering effective work-related breastfeeding interventions targeting women employed in the informal vs. the formal economy, still need to be elucidated using qualitative and quantitative implementation research approaches.

## Conclusion

Workplace breastfeeding interventions work through raising awareness among employees, supervisors and co-workers, changes in workplace breastfeeding culture, including knowledge, attitudes, and support from managers/supervisors and co-workers, and improvements in the physical environment, alongside with the time release needed by working mothers while at work for breastfeeding or extracting breastmilk. In order to better address breastfeeding inequities affecting working mothers, workplace breastfeeding interventions need to be tailored according to several contextual factors including socioeconomic and demographic characteristics of the mothers or end users. The evidence of this review clearly shows that workplace breastfeeding interventions cannot follow a one-size-fits-all approach, but rather should be tailored for the contextual factors underlying the different working conditions for mothers globally.

## Supplementary Information

**Additional file 1.** Search strategies of all searched databases.

**Additional file 2.** Publications not published in English, Spanish, Portuguese, or German.

## Data Availability

Data sharing not applicable to this article as no datasets were generated or analyzed during the current study.
