# Supplementary material for: How do breastfeeding workplace interventions work?: a realist review
Source: Int J Equity Health. 2021 Jun 25;20:148. doi: 10.1186/s12939-021-01490-7 (PMC8234653; doi:10.1186/s12939-021-01490-7)
Supplement: Supplementary file 1 — Additional file 1. Search strategies of all searched databases. [file 12939_2021_1490_MOESM1_ESM.pdf]

APPENDIX I: SEARCH STRATEGIES

Searches are provided for all the bibliographic databases. Date coverage/date searched and platforms are listed. The multiline Ovid searches can be rerun at https://tools.ovid.com/ovidtools/launcher.html by anyone with access to Medline and Global Health through Ovid. Please check whether any unsupported characters or unintended line breaks have been introduced by the typesetting and copy-pasting process.

Ovid MEDLINE(R) ALL <1946 to October 01, 2020>		
Line	Query [comments in square brackets]	Results
1	[Kathrin Litwan project]	0
2	[medline]	0
3	[breastfeeding concept]	0
4	exp breast feeding/	38035
5	(breastfe* or breast-fe*).mp.	58767
6	lactation.mp.	60214
7	breast pump*.mp.	389
8	(express* adj2 milk).mp.	1604
9	[workplace context concept]	0
10	workplace/	23180
11	Employment/ or work/ or “personnel staffing and scheduling”/	81730
12	women, working/	5375
13	return to work/	2629
14	job satisfaction/	25055
15	work schedule tolerance/	6918
16	[tight focus on breastfeeding]	0
17	3 or 4 or 5 or 6 or 7 or 8	110302
18	[for Covidence upload and screening, this is a set of papers with an explicit breastfeeding reference and EITHER workplace indexing OR *if* they haven't yet been indexed, some form of the word "work" in the title or author keywords]	0
19	10 or 11 or 12 or 13 or 14 or 15	132080
20	work*.ti,kf. not medline.st.	38425
21	17 and (19 or 20)	1007
Searched on October 2, 2020		

Web of Science Core Collection		
Indexes=SCI-EXPANDED, SSCI, A&HCI, CPCI-S, CPCI-SSH, BKCI-S, BKCI-SSH, ESCI, CCR-EXPANDED, IC Timespan=All years		
3	1107	#1 AND #2

2	938256	TI=(work*) OR AK=(work*) OR KP=(work*)
1	92705	TS=(breastfe* or breast-fe* or lactation or breast-pump* or (express* NEAR/2 milk))
Searched on November 4, 2020		

1

CINAHL (Ebsco)				
#	Query	Limiters/Expanders	Last Run Via	Results
S12	S10 AND S11	Expanders – Apply equivalent subjects Search modes – Boolean/Phrase	Interface – EBSCOhost Research Databases Search Screen – Advanced Search Database – CINAHL Complete	1307
S11	S1 OR S2	Expanders – Apply equivalent subjects Search modes – Boolean/Phrase	Interface – EBSCOhost Research Databases Search Screen – Advanced Search Database – CINAHL Complete	33887
S10	S3 OR S4 OR S5 OR S6 OR S7 OR S8 OR S9	Expanders – Apply equivalent subjects Search modes – Boolean/Phrase	Interface – EBSCOhost Research Databases Search Screen – Advanced Search Database – CINAHL Complete	243486
S9	TI(work*)	Expanders – Apply equivalent subjects Search modes – Boolean/Phrase	Interface – EBSCOhost Research Databases Search Screen – Advanced Search Database – CINAHL Complete	144324
S8	(MH "Job Satisfaction")	Expanders – Apply equivalent subjects Search modes – Boolean/Phrase	Interface – EBSCOhost Research Databases Search Screen – Advanced Search Database – CINAHL Complete	21431
S7	(MH "Personnel Staffing and Scheduling") OR (MH "Flexible Scheduling") OR (MH "Shiftwork") OR (MH "Job RE-Entry")	Expanders – Apply equivalent subjects Search modes – Boolean/Phrase	Interface – EBSCOhost Research Databases Search Screen – Advanced Search Database – CINAHL Complete	32759
S6	(MH "Women, Working+")	Expanders – Apply equivalent subjects Search modes – Boolean/Phrase	Interface – EBSCOhost Research Databases Search Screen – Advanced Search Database – CINAHL Complete	4496
S5	(MH "Work+")	Expanders – Apply equivalent subjects Search modes – Boolean/Phrase	Interface – EBSCOhost Research Databases Search Screen – Advanced Search Database – CINAHL Complete	7459
S4	(MH "Employment+")	Expanders – Apply equivalent subjects Search modes – Boolean/Phrase	Interface – EBSCOhost Research Databases Search Screen – Advanced Search Database – CINAHL Complete	46122
S3	(MH "Work Environment+") OR (MH "Job Accommodation")	Expanders – Apply equivalent subjects Search modes – Boolean/Phrase	Interface – EBSCOhost Research Databases Search Screen – Advanced Search Database – CINAHL Complete	34254

S2	(MH “Breast Feeding+”)	Search modes – Boolean/Phrase	Interface – EBSCOhost Research Databases Search Screen – Advanced Search Database – CINAHL Complete	24024
S1	TI(breastfe* or breast-fe* or lactation or brast-pump* or (express* NEAR/2 milk)) OR AB (breastfe* or breast-fe* or lactation or breast-pump* or (express* NEAR/2 milk))	Search modes – Boolean/Phrase	Interface – EBSCOhost Research Databases Search Screen – Advanced Search Database – CINAHL Complete	26241
Searched on November 4, 2020				

Global Health <1910 to 2020 Week 42>		
Line	Query [comments in square brackets]	Results
1	[Kathrin Litwan project]	0
2	[global health]	0
3	[breastfeeding concept]	0
4	exp breast feeding/ or breast pumps/ or preweaning period/	22011
5	(breastfe* or breast-fe* or lactation or breast pump* or (express* adj2 milk)).mp.	50693
6	[workplace context concept]	0
7	work places/ or exp labour/ or exp work/ or exp workshops/ or exp work satisfaction/	15765
8	exp employment/ or exp employed women/ or exp working life/	4813
9	working hours/	683
10	work*.ti.	48220
11	(4 or 5) and (7 or 8 or 9 or 10)	779
Searched on November 4, 2020		

LILACS
ti:(work* OR employ*) AND tw:(breastfe* OR "breast fed" OR "breast feed" OR "breast feeding" OR lactation OR "breast pump" OR "breast pumps" OR "breast pumping")
92 results
Search on November 18, 2020

Global Index Medicus
ti:(work* OR employ*) AND tw:(breastfe* OR "breast fed" OR "breast feed" OR "breast feeding" OR lactation OR "breast pump" OR "breast pumps" OR "breast pumping")
156 results
Search on November 18, 2020

Appendix I – How do breastfeeding workplace interventions work?: a realist review

Business Source Complete				
#	Query	Limiters/Expanders	Last Run Via	Results
S3	(S1 AND S2)	Expanders - Also search within the full text of the articles; Apply equivalent subjects<br>Search modes - Boolean/Phrase	Interface - EBSCOhost Research Databases<br>Search Screen - Advanced Search<br>Database - Business Source Complete	307
S2	TI (work* or employ*) OR SU (work* or employ* OR industrial hygiene) OR KW (work* or employ*)	Expanders - Also search within the full text of the articles; Apply equivalent subjects<br>Search modes - Boolean/Phrase	Interface - EBSCOhost Research Databases<br>Search Screen - Advanced Search<br>Database - Business Source Complete	1143114
S1	TI (breastfe* OR breast-fe* or lactation) OR AB (breastfe* OR breast-fe* or lactation) OR KW (breastfe* OR breast-fe* or lactation)	Expanders - Also search within the full text of the articles; Apply equivalent subjects<br>Search modes - Boolean/Phrase	Interface - EBSCOhost Research Databases<br>Search Screen - Advanced Search<br>Database - Business Source Complete	5168
Searched on November 11, 2020				

1

ProQuest Dissertations & Theses Global
noft(breastfe* OR breast-fe* OR lactation OR breast-pump* OR (express* NEAR/2 milk)) AND (ti(work* OR employ* or job or jobs) OR su(work* OR employ* or job or jobs))
124 results
Searched on November 11, 2020

2

Open Access Theses and Dissertations
((title:(work* OR employ*) OR subject:(work* OR employ*))) AND (breastfe* OR "breast fed" OR "breast feed" OR "breast feeding" OR lactation OR "breast pump" OR "breast pumps" OR "breast pumping")

106 results
Searched on November 11, 2020
