# Supplementary material for: How do breastfeeding workplace interventions work?: a realist review
Source: Int J Equity Health. 2021 Jun 25;20:148. doi: 10.1186/s12939-021-01490-7 (PMC8234653; doi:10.1186/s12939-021-01490-7)
Supplement: Supplementary file 2 — Additional file 2. Publications not published in English, Spanish, Portuguese, or German. [file 12939_2021_1490_MOESM2_ESM.pdf]

APPENDIX 2: PUBLICATIONS NOT PUBLISHED IN ENGLISH, SPANISH, PORTUGUESE OR GERMAN. Workplace interventions to support breastfeeding realist review.

Title	Authors	Year	Journal	Volume(Issue):Pages	DOI	Published language
Better conditions for nursing mothers in the labor market	Anonymous	1985	Sygeplejersken	85(19):12-13	-	Danish
Effects of a breast feeding promotion program for working women	Yoon, Ji-Won; Park, Young-Joo	2008	Journal of Korean Academy of Nursing	38(6): 843-852	https://dx.doi.org/10.4040/jkan.2008.38.6.843	Korean
Fostering a breastfeeding-friendly workplace	Chen, Yi-Chun; Kuo, Shu-Chen	2013	Hu li za zhi The journal of nursing	60(1):17-22	https://dx.doi.org/10.6224/JN.60.1.17	Chinese
Study on mothers return to work and supportive status of breastfeeding in workplace in Shanghai	Hu, QiaoZhen; Jiang, Hong; Li, Mu	2013	Maternal and Child Health Care of China	28(11): 1778-1781	-	Chinese
